# Does light color during brooding and rearing impact broiler productivity?

**DOI:** 10.1016/j.psj.2022.101937

**Published:** 2022-04-30

**Authors:** B.M. Remonato Franco, T. Shynkaruk, T. Crowe, B. Fancher, N. French, S. Gillingham, K. Schwean-Lardner

**Affiliations:** ⁎ Department of Animal and Poultry Science, University of Saskatchewan, Saskatoon S7N 5A8 SK, Canada; † Department of Mechanical Engineering, University of Saskatchewan, Saskatoon S7N 5A9 SK, Canada; ‡ Aviagen^TM^, 920 Explorer Blvd. NW, Huntsville, AL 35806, USA

**Keywords:** wavelength, light color, broiler, production

## Abstract

Light color during brooding and rearing may impact broiler production; however, literature results are inconsistent. To address this, the effects of 3 wavelength spectra on broiler performance in 2 sex and 2 genotypes (Ross YPMx708 and EPMx708) were studied. Broilers were raised (d 0–35) under wavelength programs provided by LED light bulbs (blue (455 nm), green (510 nm) or white) under similar intensities (clux). Two trials were conducted (total number of birds =  14,256; 6 room replications per lighting treatment; 18 replicate pens per light × sex × genotype). Data were analyzed as a 3 × 2 × 2 (wavelength × sex × genotype) factorial design, with trial as a random variable block and wavelength nested within rooms (Proc Mixed, SAS 9.4). Birds raised under white light were heavier than under blue or green light at d7 (*P* = 0.004), and green at d14 (*P* = 0.03). Feed intake, gain-to-feed efficiency and flock uniformity (d15, 28) did not differ. Mortality only differed at wk 5, when broilers raised under white light had higher mortality than those raised under blue (*P* = 0.03). YPM-708 were heavier at 21 d (*P* = 0.007), 28 d (*P* = 0.001), and 35 d (*P* < 0.0001), had a better total feed conversion rate (*P* < 0.0001), higher mortality for wk 1 (*P* = 0.001), lower mortality during the last wk (*P* = 0.02) and better uniformity at 28 d (*P* = 0.01) than EPM-708 broilers. Males were heavier at all measured ages except d0 (d7-*P* = 0.03, other weeks *P* < 0.0001), had better total feed conversion (*P* < 0.0001), increased weekly mortality except for wk 1 (wk2-*P* = 0.04, wk3-*P* = 0.002, wk4, 5, and total-*P* = 0.0001) and were less uniform (*P* = 0.0002) than females. YPM-708 and EPM-708 males had higher total feed intake (*P* < 0.0001), and males raised under white light had higher mortality than females raised under white or blue light (*P* = 0.01). To conclude, the use of specific light colors (blue and green) had only minor impacts on broiler production when light intensity was equalized and balanced for bird spectral sensitivity, and its use to improve productivity does not appear to be advantageous for broilers in a commercial setting.

## INTRODUCTION

Broiler production is impacted by many management factors, including components of lighting. Light is an important instrument used in poultry production, and variations in its programming can impact behavior, welfare, physiology, and production. Three main characteristics compose light: photoperiod (duration and distribution over 24 h), intensity, and wavelength (light color), and each of these components is capable of causing alterations to diverse aspects of broiler production (Çapar Akyüz and Onbaşilar, 2017).

Lighting programs for broilers can be designed by customizing the above-mentioned light characteristics. It is important to take into consideration the great importance of the sense of vision of birds during this customization in order to maximize the output of their species-specific behaviors, as light can impact their ability to perceive their environment (Collins et al., 2011; Wisely et al., 2017). Due to several anatomical and physiological differences, birds respond differently to light color (wavelength) compared to humans. Both species have one maximum peak of sensitivity at similar points on the spectrum (545–575 nm, corresponding to the green color). However, birds display other peaks of sensitivity in the spectrum that humans do not. These peaks are located between 400 and 480 nm, corresponding to violet/blue light, and 580 to 700 nm, corresponding to orange/red light (Lewis and Morris, 2000). Additionally, birds can perceive ultraviolet (**UV**) rays, as the presence of an extra type of cone photoreceptor and their optically clear aqueous humor and lens allow them to respond to radiation below 400 nm (Lewis and Morris, 2000; Egbuniwe and Ayo, 2016). Furthermore, the presence of oil droplets in each cone cell allows birds to discriminate colors more accurately than mammals (Stavenga and Wilts, 2014).

In birds, light can reach the brain through the eye, where photopigments, located in the cone or rod cells, absorb light in the retina, and the electrical signals formed are transmitted to the brain through the optic nerve. A second path of light reception is via the penetration of light through the skull, where light reaches the pineal gland, situated in a triangular area behind the brain, or the hypothalamus (Çapar Akyüz and Onbaşilar, 2017). In contrast to humans, the avian pineal gland functions as a circadian clock and a functional photoreceptor (Lewis and Morris, 2006). Varying wavelengths may impact the capacity of light to penetrate the retina or the skull, and penetration at various degrees may result in different behavioral expression and physiological responses. An example is an effect of red light on egg-producing hens, which results in an improvement in egg number as compared to those raised under white light. This has been attributed to a higher degree of light reception occurring mainly via the skull (Baxter et al., 2014).

There are a variety of lighting sources that can be utilized in poultry production systems. Light-emitting diode (**LED**) lamps are of interest because they are highly efficient, producing very little heat, which makes them inexpensive to operate. They can also provide specific monochromatic light colors to birds (Parvin et al., 2014). Research has been conducted on the impact of providing different monochromatic light during broiler production; however, results published to date are inconsistent. In previous experiments, raising broilers under shorter wavelengths, such as blue (460 nm) and green (560 nm), resulted in greater weight gain (Mohamed et al., 2017) and a better feed-to-gain conversion ratio (Rozenboim et al., 2004). Other authors reported that broilers raised under yellow light (580–590 nm) attained a higher body weight (Kim et al., 2013). In contrast, different studies found no impact of wavelength on broiler production (Wathes et al., 1982; Praynito et al., 1997).

To gain a better understanding of the effect of light wavelengths on broiler production, the objective of this study was to determine the impact of three light treatments, blue (455 nm), green (510 nm) and white (a combination of wavelengths), on production traits of broilers (both genders and two genotypes) raised under a simulated industry setting. The work described in this paper is part of a larger project, which includes studying of behavior, visual ability, health, and meat yield of broilers reared under these lighting conditions.

## MATERIALS AND METHODS

This experiment was approved by the Animal Care Committee of the University of Saskatchewan and was conducted following the guidelines of the Canadian Council on Animal Care (2009) as specified in the Guide to the Care and Use of Experimental Animals.

### Housing and Management

The experiment, conducted in 2 blocked trials, was designed to mimic commercial production units, using a commercial-level stocking density. The broilers were housed from 0 to 35 d, in 9 individually environmentally controlled rooms; each room was subdivided in 12 individual pens (2 × 2.3 m each). Within each room, broilers were separated based on sex and genotype (Ross YPMx708 and Ross EPMx708; 3 pens for each sex × genotype per room).

For each trial, a total of 7,128 broilers (N = 14,256 birds) were hatched at a commercial hatchery, feather sexed, transported to the research facility, and housed on the day of hatch in pens within 9 rooms. The final estimated density was calculated based on the predicted weight at d 35, with a target maximum density of 31 kg/m^2^, resulting in 62 Ross YPMx708 or EPMx708 males, or 70 Ross YPMx708 or EPMx708 females per pen. The experiment consisted of 6 room replications per lighting treatment, and 18 replicate pens per sex × genotype × lighting program.

Water and feed were available ad libitum. Birds were fed using aluminum tube feeders (110 cm of pan circumference from 0 to 30 d and 137.5cm from 30 d to market). Water was provided using pendulum nipple drinkers, with 6 nipples available per pen. Birds were fed diets obtained from a commercial feed company, formulated based on Aviagen's Ross 708 requirements (Aviagen, 2019). The starter diet (0.5 kg per bird) was provided in a crumble form, the grower (2 kg per bird) was provided in a course crumble and the finisher diet was presented in a small pellet. Supplemental feeders and drinkers were provided for wk 1.

Prior to chick placement, all pens were bedded with an equal amount of wheat straw, resulting in an approximate thickness of 7.5 to 10 cm. The temperature in the rooms was 32.1°C on d 0 and was reduced gradually (approximately 0.5°C daily) until 21°C was reached by d 25. This temperature was maintained until birds were marketed. Humidifiers were added to each room at chick placement and were removed by d 4, to maintain between 40% and 60% relative humidity during the early brooding period. Temperature and humidity monitored twice each day.

### Lighting

**Figure 1:**
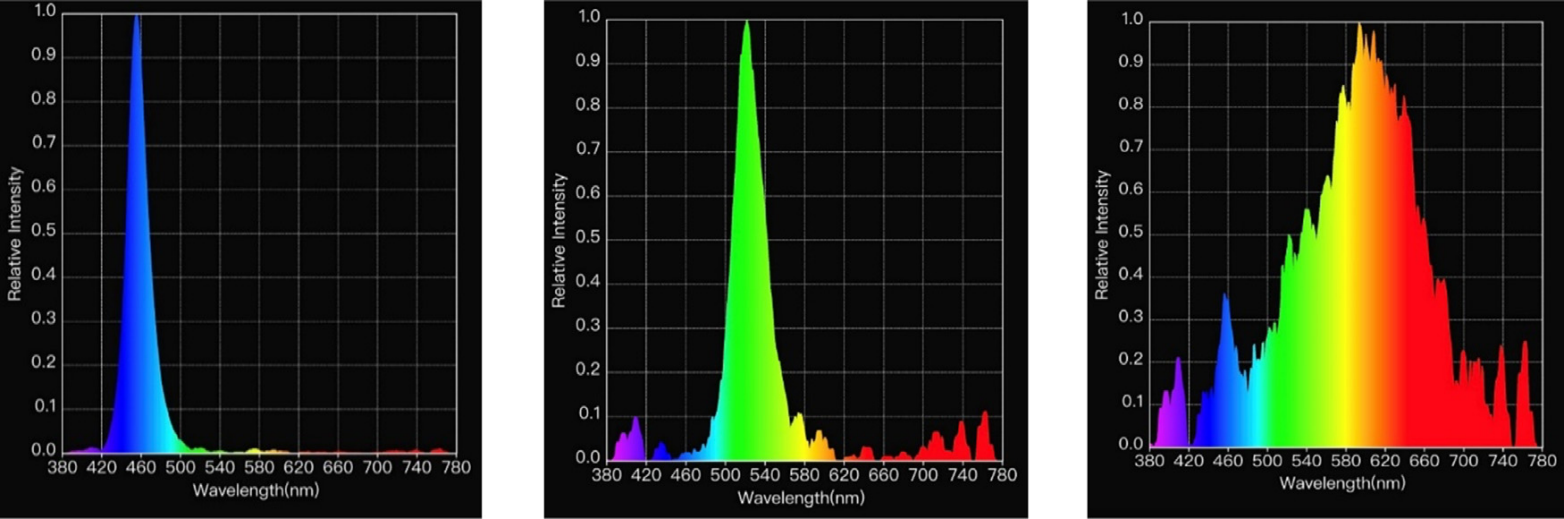
Measurements of light spectrum respectively from blue (a), green (b) and white (c) treatments.

The LED bulbs (11W Alice Non-Directional LED Lamps, Greengage Agritech Limited, Roslin Innovation Centre, University of Edinburgh, Easter Bush Campus, Midlothian, EH25 9RG, United Kingdom) provided 1 of 3 wavelength programs for the lighting treatment, consisting of colors corresponding to blue (peak at 455 nm), green (peak at 510 nm) or white. Each room's light spectrum was measured before birds were placed, using a Lighting Passport light meter (Asensetek Ιncorporation, New Taipei City, Taiwan). The measured light spectrum for each of the lighting treatments is shown in Figure 1.

The diurnal photoperiod program was started at 23L:1D for d 0, and the length of the light period was decreased by 1 h per day until it reached 18L:6D by 5 d of age. Dawn and dusk periods were set to occur for 15 min every day, once prior to lights turning on and once prior to lights turning off (included in the photophase time).

Light intensity was measured in units of clux (based on bird spectral sensitivity; Lewis and Morris, 2000), and was assessed at bird head height prior to placement (Galilux Light Meter, Hato Agricultural Lighting, Sittard, The Netherlands). For trial 1, the light intensity was 9.6 ± 0.4 clux from d 0 to 35 in all rooms. In trial 2, the intensity during the first wk was set at 14.3 ± 0.1 clux and the remaining wks at 9.6 ± 0.4 clux.

#### Data Collection

Birds were checked twice each day, and mortality and cull birds were removed and recorded. All mortality and cull birds were sent for necropsy to determine the primary cause of death or illness at an independent laboratory (Prairie Diagnosis Service [**PDS**], Western College of Veterinary Medicine, Saskatoon, Canada).

Body weight was calculated by weighing all birds on a pen basis at d 0, 7, 14, 21, 28, and 35. Feeders were weighed at the same time to allow calculation of feed intake and feed efficiency ratio (gain-to-feed ratio, with and without mortality correction). Uniformity was assessed by weighing individual birds (1 pen × genotype × sex × room) at d 14 and 28 and was expressed as the percentage of birds found within 10% and 15% of the mean.

To assess the percentage of live birds with scratches, birds were carefully handled individually (all birds in each of 1 pen × genotype × sex × room) on d 28. The presence of scratches was observed on the surface of the body, and if found, were recorded. Absence of scratches was recorded if no scratches were found.

### Statistical Analyses

Data were statistically analyzed using SAS (SAS 9.4, Cary, NC). The main effects were wavelength treatment, sex and genotype. Prior to analyses, all data were tested for normality using the UNIVARIATE procedure, and if not normally distributed, data were log-transformed to meet these assumptions. Data were analyzed with an ANOVA as a 3 (wavelength treatment) × 2 (sex) × 2 (genotype) factorial design, with light nested within room and trial as a random variable block, using the MIXED procedure. Tukey's range test was used to separate means when significant differences were found. Replicate units were room for light color, and pen for sex and genotype. Differences were considered significant when *P* < 0.05.

## RESULTS

### Body Weight and Feed Consumption

The impact of wavelength treatment, sex and genotype on body weight is shown in Table 1. No significant interactions were observed between light, genotype, or sex on body weight. A difference in body weight for broilers raised under different light colors was found at d 7, where birds raised under white light were heavier than those raised under blue or green light (*P* = 0.004). At 14 d, broilers reared under white light remained heavier than those raised under green light only (*P* = 0.03). Body weights at d 0, 21, 28 and 35 were unaffected by light treatment. As for genotypes, EPM-708 birds were heavier at 0 (*P* < 0.0001) and 7 d (*P* = 0.002), however, at 21 (*P* = 0.007), 28 (*P* = 0.001), and 35 d (*P* < 0.0001), YPM-708 birds were heavier. Males were heavier than females throughout the experiment.

**Table 1:** Effects of light color,^1^ genotype and sex, and their interactions, on broiler body weight (kg) and feed intake (kg/bird) at 0–7, 7–14, 14–21, 21–28, and 28–35 d of age

	Light				Genotype			Sex			Interactions				
	Blue	Green	White	*P* value	Y-708	E-708	*P* value	Male	Female	*P* value	Light ×genotype	Light× sex	Genotype× sex	Light ×genotype× sex	SEM^2^
Weight (kg)															
*0d*	0.039	0.039	0.039	0.91	0.038^b^	0.039^a^	<0.0001	0.039^a^	0.038^b^	0.01	0.27	0.61	0.93	0.41	0.001
*7d*	0.164^b^	0.164^b^	0.166^a^	0.004	0.164^b^	0.166^a^	0.002	0.165^a^	0.164^b^	0.03	0.70	0.47	0.36	0.66	0.001
*14d*	0.482^ab^	0.480^b^	0.489^a^	0.03	0.484	0.484	0.54	0.495^a^	0.473^b^	<0.0001	0.51	0.79	0.59	0.66	0.002
*21d*	1.018	1.064	1.030	0.49	1.042^a^	1.032^b^	0.007	1.080^a^	0.994^b^	<0.0001	0.06	0.60	0.43	0.89	0.006
*28d*	1.706	1.708	1.716	0.67	1.719^a^	1.701^b^	0.001	1.796^a^	1.624^b^	<0.0001	0.19	0.86	0.08	0.60	0.008
*35d*	2.457	2.447	2.464	0.77	2.488^a^	2.430^b^	<0.0001	2.594^a^	2.318^b^	<0.0001	0.11	0.51	0.58	0.65	0.012
Feed intake (kg/bird)															
*0-7d*	0.143	0.143	0.145	0.20	0.144	0.143	0.67	0.145^a^	0.142^b^	0.0004	0.31	0.25	0.08	0.58	0.001
*7-14d*	0.405	0.408	0.410	0.59	0.406^b^	0.410^a^	0.03	0.414^a^	0.402^b^	<0.0001	0.52	0.44	0.54	0.22	0.002
*14-21d*	0.725	0.723	0.730	0.21	0.732^a^	0.718^b^	<0.0001	0.761^a^	0.690^b^	<0.0001	0.52	0.90	<0.0001	0.94	0.003
*21-28d*	0.992	1.003	1.005	0.60	0.999	1.001	0.68	1.046^a^	0.955^b^	<0.0001	0.10	0.56	0.64	0.76	0.004
*28-35d*	1.268	1.256	1.264	0.67	1.255^b^	1.270^a^	0.02	1.333^a^	1.192^b^	<0.0001	0.12	0.51	0.38	0.38	0.006
*0-35d*	3.533	3.533	3.554	0.46	3.538	3.542	0.72	3.699^a^	3.381^b^	<0.0001	0.06	0.52	<0.0001	0.84	0.020
*Interactions between genotype and sex on feed intake*															
	Y-708 Male		Y-708 Female		E-708 Male		E-708 Female								
*14-21d*	0.760^a^		0.659^c^		0.711^b^		0.655^c^								
*0-35d*	3.786^a^		3.286^c^		3.635^a^		3.261^b^								

1 Dominant wavelengths for the blue treatment ranged from 435 to 500 nm, while the green treatment was dominated by 500–565 nm, and a combination of wavelengths produced white light.

2 SEM, Standard error of the mean.

a,b Means with common letters in the same row do not differ significantly (*P* ≤ 0.05).

No impact of lighting treatment was found on feed consumption. EPM-708 birds consumed more feed from d 7 to 14 (*P* = 0.03) and 28 to 35 (*P* = 0.02). Males consumed more feed than females. Interactions were noted between genotype and sex for the d 14 to 21 and 0 to 35 d periods, where, from 14 to 21 d, YPM-708 male birds ate more than YPM-708 females and EPM-708 birds and YPM-708 and EPM-708 males consumed more feed from 0 to 35 d.

### Feed Conversion Ratio

Results related to feed conversion ratio (gain-to-feed and gain-to-feed corrected for mortality) are presented in Table 2. Light color did not impact feed conversion ratio. From d 28 to 35 (*P* < 0.0001) and overall (*P* < 0.0001), YPM-708 birds were more feed efficient when data were corrected for mortality, but if mortality was not considered, EPM-708 birds were more efficient from d 0 to 7 (*P* = 0.04). Males had a better gain-to-feed ratio when corrected for mortality than females, except during the d 0 to 7 period. For the gain-to-feed ratio without mortality correction, interactions were noted between genotype and sex, where YPM-708 males had reduced feed efficiency from d 14 to 21, and YPM-708 and EPM-708 females had better feed efficiency overall.

**Table 2:** Effects of wavelength,^1^ genotype, and sex, and their interactions on gain-to-feed ratio, with and without mortality correction at 0–7, 7–14, 14–21, 21–28, and 28–35 d of age

	Light				Genotype			Sex			Interactions				
	Blue	Green	White	*P* value	Y-708	E-708	*P* value	Male	Female	*P* value	Light × genotype	Light× sex	Genotype× sex	Light ×genotype× sex	SEM^2^
*G:F_m_*															
*0–7d*	0.967	0.969	0.971	0.88	0.965	0.972	0.08	0.964^b^	0.974^a^	0.01	0.93	0.08	0.24	0.43	0.002
*7–14d*	0.828	0.831	0.830	0.92	0.832	0.827	0.33	0.845^a^	0.814^b^	<0.0001	0.65	0.13	0.65	0.24	0.002
*14–21d*	0.776	0.873	0.777	0.39	0.811	0.807	0.25	0.820^a^	0.798^b^	<0.0001	0.43	0.96	0.66	0.77	0.008
*21–28d*	0.707	0.651	0.700	0.43	0.687	0.685	0.75	0.696^a^	0.676^b^	0.0007	0.45	0.25	0.22	0.58	0.005
*28–35d*	0.619	0.616	0.618	0.96	0.629^a^	0.606^b^	<0.0001	0.682^a^	0.607^b^	<0.0001	0.75	0.87	0.92	0.29	0.002
*Total*	0.715	0.715	0.714	0.97	0.720^a^	0.710^b^	<0.0001	0.726^a^	0.704^b^	<0.0001	0.39	0.77	0.06	0.27	0.001
*G:F*															
*0–7d*	0.967	0.970	0.968	0.92	0.964^b^	0.973^a^	0.04	0.964^b^	0.973^a^	0.03	0.96	0.16	0.29	0.39	0.002
*7–14d*	0.835	0.828	0.833	0.76	0.835	0.829	0.12	0.844^a^	0.820^b^	<0.0001	0.63	0.24	0.78	0.39	0.002
*14–21d*	0.742	0.751	0.751	0.65	0.721^b^	0.775^a^	<0.0001	0.732^b^	0.764^a^	0.001	0.93	0.88	<0.0001	0.95	0.005
*21–28d*	0.688	0.684	0.682	0.92	0.679	0.691	0.11	0.682	0.687	0.51	0.18	0.47	0.34	0.58	0.003
*28–35d*	0.592	0.568	0.574	0.54	0.584	0.573	0.14	0.559^b^	0.598^a^	<0.0001	0.42	0.39	0.58	0.72	0.004
*Total*	0.694	0.688	0.689	0.61	0.687^b^	0.694^a^	0.02	0.682^a^	0.699^b^	<0.0001	0.15	0.80	0.0005	0.58	0.002
*Interactions between genotype and sex on G:F*															
	Y-708 Male		Y-708 Female		E-708 Male		E-708 Female								
*G:F 14–21d*	0.693^b^		0.794^a^		0.810^a^		0.790^a^								
*G:F total*	0.682^b^		0.700^a^		0.693^b^		0.692^a^								

1 Dominant wavelengths for the blue treatment ranged from 435 to 500 nm, while the green treatment was dominated by 500–565 nm, and a combination of wavelengths produced white light.

2 SEM = Standard error of the mean.

ab Means with common letters in the same row do not differ significantly (*P* ≤ 0.05).

### Mortality

Light color had no effect on the percentage of mortality observed, except for the period between 28 and 35 d (*P* = 0.03), when mortality was higher in birds raised under white light as compared to blue (1.76% vs. 1.08% respectively, Table 3). An interaction between lighting treatment and sex was noted over d 0 to 35, where males raised under white light had higher mortality than females raised under blue, green, or white light (*P* = 0.01). Between 0 and 7 d, YPM-708 birds had higher mortality than EPM-708 broilers (*P* = 0.001), while between 28 and 35 d, the mortality level of YPM-708 birds was lower than EPM-708 broilers (*P* = 0.02). Males had higher mortality than females (d 7–14, *P* = 0.04, d14-21, *P* = 0.002), d21 to 28, *P* = 0.0001, d28-35, *P* < 0.0001, d0-35, *P* < 0.0001).

**Table 3:** Effects of light color,^1^ genotype and sex, and their interactions, on broiler mortality (%) from 0–7, 7–14, 14–21, 21–28, and 28–35 d of age.

	Light				Genotype			Sex			Interaction *P* values				
	Blue	Green	White	*P* value	Y-708	E-708	*P* value	Male	Female	*P* value	Light ×genotype	Light ×sex	Genotype× sex	Light x genotype× sex	SEM^2^
*0-7d*	1.42	1.80	1.64	0.76	2.13^a^	1.11^b^	0.001	1.68	1.56	0.71	0.96	0.43	0.70	0.12	0.177
*7-14d*	1.20	1.24	1.26	0.97	1.38	1.09	0.16	1.45^a^	1.02^b^	0.04	0.94	0.24	0.44	0.11	0.109
*14-21d*	1.18	0.89	0.94	0.42	1.14	0.87	0.14	1.28^a^	0.72^b^	0.002	0.43	0.22	0.87	0.96	0.091
*21-28d*	1.12	1.08	1.00	0.63	0.96	1.24	0.10	1.43^a^	0.77^b^	0.0001	0.06	0.10	0.92	0.80	0.090
*28-35d*	1.08^b^	1.44^ab^	1.76^a^	0.03	1.19^b^	1.66^a^	0.02	2.14^a^	0.71^b^	<0.0001	0.21	0.31	0.31	0.45	0.116
*0-35d*	6.01	6.45	6.59	0.74	6.76	5.94	0.08	7.95^a^	4.76^b^	<0.0001	0.11	0.01	0.87	0.13	0.291
*Interactions between light x sex on mortality levels*															
	Blue - Male		Blue - Female		Green - Male		Green - Female		White - Male		White - Female				
*0-35 d*	1.57^ab^		0.60^cd^		2.24^ab^^c^		0.63^b^^cd^		2.60^a^		0.91^d^				

1 Dominant wavelengths for the blue treatment ranged from 435 to 500 nm, while the green treatment was dominated by 500–565 nm, and a combination of wavelengths produced white light.

2 SEM, Standard error of the mean.

a,b Means with common letters in the same row do not differ significantly (*P* ≤ 0.05)

### Uniformity

Light color did not impact flock uniformity at 14 or 28 d (Table 4). YPM-708 broilers were more uniform than EPM-708 broilers at 28 d (*P* = 0.002 and *P* = 0.01) and females were more uniform than males at both 14 (*P* < 0.0001 and *P* = 0.0008) and 28 d (*P* < 0.0001 and *P* = 0.0002). No interactions between light color, genotype and sex were found.

**Table 4:** Effects of different light colors^1^ on broiler uniformity expressed as the percentage of birds within 10 and 15% of the mean body weight at 14 and 28 days

	Light				Genotype			Sex			Interactions			
	Blue	Green	White	*P* value	Y-708	E-708	*P* value	Male	Female	*P* value	Light ×genotype	Light× sex	Genotype × sex	Light × genotype × sex
*14 d (% in the range around mean)*														
10%	75.49	72.82	73.57	0.42	75.30	72.61	0.10	70.23^b^	77.68^a^	<0.0001	0.52	0.89	0.96	0.61
15%	90.82	88.65	89.39	0.47	90.35	88.90	0.22	87.49^b^	91.75^a^	0.0008	0.30	0.74	0.99	0.29
*28 d (% in the range around mean)*														
10%	74.44	72.48	70.28	0.10	74.87^a^	69.94^b^	0.002	67.82^b^	76.99^a^	<0.0001	0.37	0.34	0.64	0.25
15%	89.40	87.66	87.82	0.41	89.84^a^	86.74^b^	0.01	85.93^b^	90.66^a^	0.0002	0.29	0.79	0.56	0.06

1 Dominant wavelengths for the blue treatment ranged from 435 to 500 nm, while the green treatment was dominated by 500–565 nm, and a combination of wavelengths produced white light.

ab Means with common letters in the same row do not differ significantly (*P* ≤ 0.05).

### Scratches

Results for the presence of scratches on live birds are shown in Table 5. Light color, genotype and sex did not impact the percentage of birds with scratches. No significant interactions between light treatment, genotype and sex were found.

**Table 5:** The effect of light color^1^, genotype and sex, and their interactions on the percentage of birds with scratches on d 28

	Light				Genotype			Sex			Interactions				
	Blue	Green	White	*P* value	Y-708	E-708	*P* value	Male	Female	*P* value	Light ×genotype	Light× sex	Genotype× sex	Light ×genotype× sex	SEM^2^
% of birds	2.89	3.09	3.06	0.91	2.98	3.05	0.54	2.95	3.06	0.21	0.07	0.52	0.27	0.11	0.046

1 Dominant wavelengths for the blue treatment ranged from 435 to 500 nm, while the green treatment was dominated by 500–565 nm, and a combination of wavelengths produced white light.

2 SEM, Standard error of the mean.

## DISCUSSION

### Light Color (Wavelength)

Light is an important instrument used in broiler production that can impact growth rate, activity levels, behavior and other characteristics (Lewis and Morris, 1998). With the switch to LED lighting, providing narrow-band monochromatic light is facilitated, and as a result, more research is being published in this area. However, the results published on the impacts of raising broilers under different wavelengths, including effects on production levels, are inconsistent (Wathes et al., 1982; Prayitno et al., 1997; Rozenboim et al., 1999; Karakaya et al., 2009; Mohamed et al., 2017). Because of these discrepancies between results, producers may find it difficult to decide on lighting choices, and clarification on the effects of different light colors (dominant wavelengths) on broiler production will facilitate this decision-making process.

In this study, we measured the effects of raising broilers under blue, green and white light treatments on production, including body weight, feed intake, feed conversion ratio, mortality levels, uniformity and the presence of scratches on live birds. Because of different visual capabilities of birds under various light colors, light intensity was equalized across treatments by measuring and adjusting using clux (corrected lux), rather than lux, which differs from many previous research articles. Once that factor was equalized across lighting treatment, color, including blue (dominant peak at 455 nm), green (peak at 510 nm) or white had inconsequential impacts on body weight at d 7 and 14 and minor impacts on the remaining production indexes that were assessed. This agreed with previous research conducted by Wathes et al. (1982) involving a larger number of birds (N = 6400) as compared to other studies, where no effect on broiler growth was found when raised under blue, green, red or white light. Likewise, Prayitno et al. (1997) found that raising broilers in blue, green, red, or white colors resulted in more evident influences on animals’ behavior rather than growth.

However, other studies have reported differences in production levels when light wavelength was tested. Previous research (Rozenboim et al., 1999; Cao et al., 2008) found an early increase in body weight when broilers were raised under green light and a later increase when broilers were raised under blue light, as compared to broilers raised under white and red light. Mohamed et al. (2017) found that birds raised under blue and green light had heavier final body weights as compared to those raised under white light. Other studies report that a combination of wavelengths treatments may also be beneficial for production levels, such as using green light early on and blue light at a later stage (Rozenboim et al., 2004; Karakaya et al., 2009; Cao et al., 2012). According to these studies, different wavelengths appear to stimulate the proliferation of muscle satellite cells, which will lead to higher muscle weight (Halevy et al., 1998). Additionally, alterations in androgen production are mentioned as a possible cause of variation in body weight, as blue and green light influence the synthesis of testosterone, leading to the promotion of myofiber growth (Cao et al., 2008).

Furthermore, results of previous studies also disagree on the effects of light wavelength on other production indexes. While feed efficiency appeared to be influenced by light wavelength in some studies (Rozenboim et al., 1999; Karakaya et al., 2009; Mohamed et al., 2017), no impact was found in the current study, which has also been found in some previous research (Wathes et al., 1982; Prayitno et al., 1997; Cao et al., 2008). Mortality appears not to be influenced by light wavelength (Rozenboim et al., 1999; Cao et al., 2008), which was supported by the current study, however mortality was not assessed in many of the previous studies. Likewise, uniformity is a parameter not assessed in previous light wavelength studies. The results of the current study showed that light wavelength does not appear to impact uniformity levels in broilers.

Relatively little is known about causes of scratches in broilers with respect to light wavelength (Villarroel et al., 2018). Previous studies describe the impact of short wavelength, such as blue light, on activity levels, where birds raised under these light colors show increased resting behavior (Prayitno et al., 1997). Previous studies also indicate the impact of short wavelength treatments on stress and fear levels, leading to calmer birds (Prayitno et al., 1997; Mohamed et al., 2014). A similar result was found by our group, where birds raised under blue light had decreased fear and stress levels when compared to broilers raised under white or green lights (Remonato Franco et al., 2019). In this study it was hypothesized that lower levels of stress and fear would reduce levels of restlessness, therefore reducing the incidence of scratches. However, no effects were found on the percentage of birds with scratches. This may indicate that factors other than stress and fear may be more related to the incidence of scratches in live birds, such as stocking density and method of catching prior to slaughter.

Inconsistencies in results reported by previous research that examined the impacts of wavelength on broiler production may be due to confounding effects of light wavelength with other factors, such as light intensity, photoperiod, light source, sample size, time of exposure to the light source, management of birds and environmental factors. It is known that light intensity may have impacts on production (Deep et al., 2013). It is also known that measuring light intensity in lux for monochromatic lighting results in incorrect values with respect to how a bird sees (Prescott and Wathes, 1999). To correct for this in research focusing on wavelength, the use of “clux,” which is a unit that accounts for bird specific spectral sensitivity, appears to be a more reliable unit of measurement (Lewis and Morris, 2006). Previous research (Rozenboim et al., 1999; Cao et al., 2008, 2012; Karakaya et al., 2009; Mohamed et al., 2017) equalized light intensity using lux, which is a common method of assessing light intensity for poultry and is based upon the spectral sensitivity of mammals. However, if lux is used, then birds may perceive the intensity as brighter or dimmer than what is expected (Lewis and Morris, 2000), and results obtained may have been affected by an interaction between wavelength and light intensity. This may help explain the discrepancy between results found in literature as compared to the current study.

Previous studies may also have a confounding effect of utilizing various light sources, ranging from incandescent and LED in the same experiment (Rozenboim et al., 1999; Karakaya et al., 2009) to LED for all treatments (Cao et al., 2008, 2012; Mohamed et al., 2017). Spectral distribution of white light may differ depending on the light source used, as each source may have a characteristic curve of wavelength emission (Archer, 2015). Incandescent light bulbs produce light through heat, and the distribution of wavelength is broad; however, they proportionally generate more red light as compared to sunlight, and they also emit infrared light (Lewis and Morris, 1998). LED lamps produce light in a narrow range of dominant wavelengths. When compared to white light produced by fluorescent bulbs, white light emitted by LED bulbs contains more blue light (Archer, 2015; Baleja et al., 2015). Furthermore, the use of LED bulbs seems to be beneficial when monochromatic colors are used, as the spectrum emitted is very narrow. If the lamps used in previous studies did not provide narrow-band monochromatic light, the impacts observed may have been influenced by other light wavelengths present in the light source. Due to the different mechanisms of emission of light by different light sources, it is also critical that the spectrum output is assessed in the barn at bird height, which was performed in the current study, to confirm that the measured output aligns with the expected output.

The photoperiod program used in each study should also be considered. Several differences between light programs were found throughout previous research, where a photoperiod of 23L:1D was common (Rozenboim et al., 1999; Cao et al., 2008, 2012; Mohamed et al., 2017). The current experiment provided broilers with 16L:8D after d 6, which is an optimal program from a welfare and performance standpoint (Schwean-Lardner et al., 2012). Other aspects of previous experiments, such as sample size, management, and environmental factors, could also have influenced the results obtained. The current study aimed to use a larger sample size as compared to previous research.

### Genotype and Sex

YPM-708 and EPM-708 broilers demonstrated slight differences in their production indexes, noted by differences in body weight after d 21, feed-conversion ratio and uniformity at d 28 and mortality during the final week. This is likely a result of the genetic selection performed by the primary breeding company on the different genotypes. An interaction was noted between genotypes and sex, where YPM-708 and EPM-708 males showed higher total feed intake compared to YPM-708 and EPM-708 females.

As expected, sex also demonstrated differences. Males were heavier and had improved feed conversion than females. Males had higher weekly mortality, except for the first wk, and were less uniform than females. An interaction was found between light and sex for total mortality, where males raised under white light had higher mortality than females raised under white or blue lights. Differences in production performance between sex are expected, and are in accordance with previous work (Schwean-Lardner et al., 2012).

## CONCLUSION

The objective of this research was to test the impact of light color on broilers by simulating a commercial setting, in a controlled environment, with significant sample size and controlled factors, such as light source and intensity. It was observed that using light with different wavelength distributions (colors) resulted in minor impacts on performance. Therefore, the application of monochromatic blue and green light in broiler production does not appear to be advantageous for productivity when light intensity and light source are controlled.
